# Impact of Vortioxetine on Inflammatory Biomarkers and Quality of Life in Patients with Chronic Heart Failure and Comorbid Depression: A Comparative Analysis of CRP, TNF-α Levels, and Functional Outcomes

**DOI:** 10.1192/j.eurpsy.2025.853

**Published:** 2025-08-26

**Authors:** A. K. Sikora, O. Pityk, S. Fedorov

**Affiliations:** 1 Psychiatry, Addictions and Medical Psychology Department, Ivano-Frankivsk National Medical University, Ivano-Frankivsk, Ukraine; 2 HOSPITAL OF THE MAZOWIECKIE VOIVODESHIP DREWNICA, Warsaw, Poland; 3 Therapy and Family Medicine Chair of Postgraduate Faculty, Ivano-Frankivsk National Medical University, Ivano-Frankivsk, Ukraine

## Abstract

**Introduction:**

Chronic heart failure (CHF) is a progressive condition often accompanied by comorbid depression, which exacerbates the overall disease burden and worsens clinical outcomes. Inflammatory processes play a crucial role in the pathophysiology of both CHF and depression. Biomarkers such as C-reactive protein (CRP) and tumor necrosis factor-alpha (TNF-α) are known to be elevated in these conditions. Vortioxetine, a multimodal antidepressant, is recognized for its efficacy in treating depression, but its impact on inflammatory markers and heart failure outcomes remains underexplored.

**Objectives:**

1. To assess the impact of Vortioxetine on CRP levels in patients with CHF and comorbid depression.
2. To evaluate the effect of Vortioxetine on TNF-α levels in the same patient population.
3. To measure the improvements in quality of life and functional outcomes using the MLHFQ and KCCQ scales.
4. To determine the significance of the observed changes in both biomarker levels and functional outcomes.

**Methods:**

- Patients: A cohort of 30 patients with chronic heart failure and comorbid depression was selected. Baseline inflammatory biomarker levels and functional outcomes were recorded.
- Intervention: All patients were treated with Vortioxetine at a dose of 10 mg/day for six months.
- Biomarkers: CRP and TNF-α levels were measured before and after six months of treatment.
- Functional Outcomes: MLHFQ, and functional status was evaluated using the KCCQ. Both assessments were conducted before and after the treatment period.

**Results:**

Biomarkers:

- CRP: Baseline CRP levels ranged from 2 mg/L to 256 mg/L, with a mean of [89mg/L]. After six months of treatment, CRP levels significantly decreased, ranging from [2mg/L], with a mean post-treatment CRP level of [10,2mg/L].
- TNF-α: Baseline TNF-α levels ranged from 0 pg/mL to 523 pg/mL, with a mean of [118pg/mL]. After treatment, TNF-α levels showed a significant reduction, with a range of [0pg/mL] and a mean post-treatment level of [18,9pg/mL].

Functional Outcomes:

- MLHFQ Scores: The mean MLHFQ score before treatment was [67], which improved by 54% after six months of Vortioxetine treatment.
- KCCQ Scores: The mean KCCQ score before treatment was [12], which improved by 46% after treatment.

**Conclusions:**

Vortioxetine has demonstrated significant potential in treating both the mental and physical aspects of chronic heart failure and comorbid depression. The observed reductions in CRP and TNF-α levels, along with the notable improvements in MLHFQ and KCCQ. scores, highlight its ability to address both inflammatory processes and patient-reported outcomes. Further research is needed to explore the long-term effects of Vortioxetine in larger patient populations and to better understand the mechanisms underlying its anti-inflammatory properties.

**Disclosure of Interest:**

None Declared

